# Partitioning of genomic variance reveals biological pathways associated with udder health and milk production traits in dairy cattle

**DOI:** 10.1186/s12711-015-0132-6

**Published:** 2015-07-14

**Authors:** Stefan M. Edwards, Bo Thomsen, Per Madsen, Peter Sørensen

**Affiliations:** Center for Quantitative Genetics and Genomics, Department of Molecular Biology and Genetics, Aarhus University, Blichers Allé 20, P.O. Box 50, Tjele, DK-8830 Denmark; Department of Molecular Biology and Genetics, Aarhus University, Blichers Allé 20, P.O. Box 50, Tjele, DK-8830 Denmark

## Abstract

**Background:**

We have used a linear mixed model (LMM) approach to examine the joint contribution of genetic markers associated with a biological pathway. However, with these markers being scattered throughout the genome, we are faced with the challenge of modelling the contribution from several, sometimes even all, chromosomes at once. Due to linkage disequilibrium (LD), all markers may be assumed to account for some genomic variance; but the question is whether random sets of markers account for the same genomic variance as markers associated with a biological pathway?

**Results:**

We applied the LMM approach to identify biological pathways associated with udder health and milk production traits in dairy cattle. A random gene sampling procedure was applied to assess the biological pathways in a dataset that has an inherently complex genetic correlation pattern due to the population structure of dairy cattle, and to linkage disequilibrium within the bovine genome and within the genes associated to the biological pathway.

**Conclusions:**

Several biological pathways that were significantly associated with health and production traits were identified in dairy cattle; i.e. the markers linked to these pathways explained more of the genomic variance and provided a better model fit than 95 % of the randomly sampled gene groups. Our results show that immune related pathways are associated with production traits, and that pathways that include a causal marker for production traits are identified with our procedure.

We are confident that the LMM approach provides a general framework to exploit and integrate prior biological information and could potentially lead to improved understanding of the genetic architecture of complex traits and diseases.

**Electronic supplementary material:**

The online version of this article (doi:10.1186/s12711-015-0132-6) contains supplementary material, which is available to authorized users.

## Background

Genome-wide association studies (GWAS) focus on determining which genes or mutations contribute to a given trait or disease phenotype by looking at each genetic variant one at a time [1]. In this approach, it is assumed that the trait or disease phenotype is inherited in a Mendelian fashion. However, in animal breeding the objective is to use all available genetic variants simultaneously to predict breeding values, since the traits of interest are complex traits such as body weight, disease susceptibility, or production of milk. The current understanding of complex traits is that the phenotype is expressed as a result of the interplay between molecular and cellular components, each component interacting at various levels (e.g. cell, molecule, RNA, DNA) that are entangled in various biological pathways. This fits well with an infinitesimal model, for which it is assumed that a large number of genetic variants contribute to the trait, each with a small effect.

Issues on how complex traits are regulated give rise to the problem of ‘missing heritability’ [2]. This problem occurs when the objective is to estimate how much of the genomic variance can be explained by the genetic variants identified in a traditional GWAS, e.g. in which the genetic variants are tested individually. After accounting for non-genetic effects (e.g. sex and environmental effects) for an observed phenotype, and adjusting for multiple testing, the genetic variants that are determined to be significantly associated cannot explain all the observed genomic variance. Makowsky et al. [3] lists some explanations such as poor genetic models (e.g. unaccounted epistatic effects), insufficient sample sizes or rare genetic variants, but highlights the work of Yang et al. [4], who suggest that the statistical modelling framework does not match the genetic architecture.

Evidence collected across numerous GWAS reveals patterns that provide insight into the genetic architecture of complex traits [5, 6]. Although many genetic variants with small or moderate effects contribute to the overall genetic variation, it appears that the genetic variants associated with trait variation are preferentially located in genes that are connected within biological pathways [5].

These biological findings are useful to improve statistical models that aim at associating genetic variation with the phenotypes of interest. There are several statistical modelling approaches that can be used to evaluate the collective action of multiple genetic variants within biological pathways or other genomic features (e.g. regulatory elements or genes) [7–10]. A commonly used two-step approach is the SNP (single nucleotide polymorphism) set enrichment analysis [7]. In this approach, the first step consists in using SNPs as proxies for causal genetic variants to obtain test statistics (e.g. t-statistics or p-values) from traditional single-marker or all-marker statistical models for the association between a phenotype and individual markers. In the second step, for each genomic feature being tested, an enrichment score is calculated (e.g. an overrepresentation of associated markers in the genomic feature). SNP set enrichment analysis is commonly used because it is computationally fast and is easily combined with association data from previous GWAS. The downside of these approaches is that they typically only use the highest scoring SNPs, thus neglecting markers that potentially each contribute very small effects [10].

The linear mixed model approach proposed by Yang et al. [4] and Jensen et al. [11] circumvents this issue by partitioning the genome according to chromosomes and then modelling the markers in each chromosome concurrently. Using this approach, they were able to determine the proportion of genomic variance that is explained by markers on each chromosome. One major advantage of this modelling approach is that it does not require a cut-off of p-values, but instead it considers all markers, whether they have a large or small effect. This is consistent with the idea that a complex trait is caused by multiple markers with small to moderate effects.

**Fig. 1:**
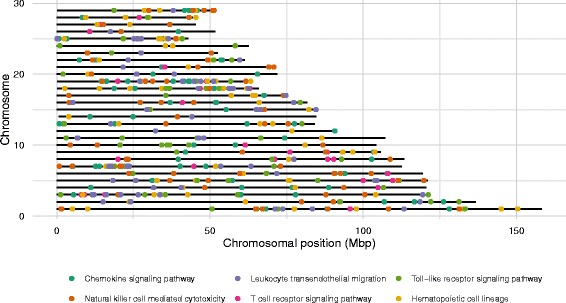
Chromosomal location of genes associated with the KEGG pathway Immune System. The pathway consists of several sub-pathways and genes that can be associated to none, one or several pathways. Since the chromosomal location of the genes is known, it is possible to link a pathway to a set of markers

To enhance the analysis and biological interpretation, instead of partitioning according to the physical structuring of genes (i.e. chromosomes), we applied external information, evidence that was obtained from other experiments or from other organisms through homology, to the partitioning concept; here we used the Kyoto Encyclopedia of Genes and Genomes (KEGG) [12, 13]. Thus, we were able to answer questions such as ‘How much of the observed phenotypic variance is accounted for by markers linked to a specific biological pathway?’ Although it is straightforward to determine a variance component for a group of markers or a pathway of interest, it is not so easy to determine whether it represents a significant amount of genomic variance. The linear mixed model approach allows us to use a likelihood ratio test (LRT) to compare different pathway-based partitionings of the genomic variance. However, genes and markers that can be associated to biological pathways are scattered throughout the genome (Fig. 1) and this may influence the distribution of the likelihood ratios. Furthermore, under the infinitesimal model, we expect that an infinite number of markers contribute to the observed genomic variance, and therefore we must also determine whether the variance contributed by the markers of interest is larger than that of the same number of randomly sampled markers.

The aims of this study were to: (1) implement a linear mixed model approach to quantify the collective action of multiple markers within biological pathways on a complex trait, (2) to evaluate testing strategies to assess whether the proportion of explained genomic variance by a set of ‘scattered’ markers is statistically significant and larger than the proportion explained by a randomly sampled set of markers, and (3) to apply this modelling approach to identify biological pathways associated with udder health and milk production traits in dairy cattle.

## Methods

### Genotype data

Genotype data for 4497 Danish Holstein bulls was obtained using Illumina BovineHD (777 k) and BovineSNP50 (50k) SNP arrays. The 50 k genotype data was imputed to the HD level using BEAGLE [14]. For more details see [15]. After filtering for markers with a minimum allele frequency greater than 0.01, 637 951 SNPs were available for analysis.

### Trait data

Records for udder health and production traits were available for 4497 bulls and are summarised in Table 1. Health traits were estimated breeding values (EBV) for clinical mastitis from 15 days before to 50 days after first calving (‘Mastitis 1.1’), clinical mastitis from 51 to 305 days after first calving (‘Mastitis 1.2’), average somatic cell score during the period between 5 amd 305 days after first calving (‘Somatic Cell Score 1’), and a composite index ‘Udder-health’ [16]. Production traits were ‘Fat yield’, ‘Milk yield’, and ‘Protein yield’, expressed as deregressed proofs (DRP) of trait indices during lactation periods after first, second, and third calving. See [16] for further description of the traits. DRP were based on EBV and associated reliabilities from routine genetic evaluations conducted by Nordic Cattle Genetic Evaluation [16]. Deregression was performed by the iterative procedure of [17] implemented in MiX99 [18, 19].

**Table 1:** Summary of the traits analysed

Trait		Number of observations	Average (Std.dev)	Record type	Random gene groups	Pathways
*Health traits*						
	Mastitis 1.1	4491	95.8 (9.7)	EBV	5553	150
	Mastitis 1.2	4394	96.3 (9.7)	EBV	5557	150
	Somatic Cell Score	4492	96.8 (10)	EBV	5550	150
	Udder-health	4497	96.1 (9.6)	EBV	5551	150
*Production traits*						
	Fat yield	4398	97.0 (12)	DRP	5591	149
	Milk yield	4398	97.4 (13)	DRP	5592	150
	Protein yield	4398	95.4 (15)	DRP	5596	148

EBV: Estimated Breeding Values; DRP: Deregressed Proof; Std.dev: Standard deviation

### Mapping pathways to genes to markers

The SNPs on the HD array are mapped to the UMD3.1 Bovine Genome assembly [20]; gene maps for this assembly were downloaded September 1st 2011 from the website ftp://ftp.cbcb.umd.edu/pub/data/assembly/Bos_taurus/Bos_taurus_UMD_3.1/annotation/UMD3.1.gff.gz, containing 26 352 genes with an Entrez Gene ID. A marker was associated with a gene if the chromosomal position of the marker was between the start and stop chromosomal position of the gene. We attempted to determine the maximum possible distance between marker and gene for a marker to be associated with a gene for distances ranging from 0 to 50 kbp (both upstream and downstream) by calculating variance components as described in the section ‘Linear mixed models’. Comparison between the proportion of explained genomic variance and distance gave no definitive conclusion, so we arbitrarily chose not to include markers located outside a gene. This resulted in 39 % (251 436/637 951) of the SNPs being mapped to 17 664 genes. Although on average there were 14 SNPs per gene, 3676 of the genes had only a single mapped SNP. At the other extreme, 369 genes had more than 100 SNPs mapped to them, with one gene having 645 SNPs mapped to it.

KEGG pathways were obtained from the KEGG’s website http://www.genome.jp/kegg-bin/get_htext?org_name=br08901&htext=br08901.keg&hier=2 and manually curated into a map-file that describes the pathways in the sections Metabolism, Genetic Information Processing, Enviromental Information Processing, Cellular Processes, and Organismal Systems. Associations between KEGG pathways and genes were extracted from the database file of the BioConductor package ‘org.Bt.eg.db’ v. 2.6.4 [21, 22]. This resulted in 4490 genes associated to KEGG pathways. A SNP was associated with a pathway if it resided within a gene linked to the pathway, and was only associated once per pathway.

SNPs located in genes that were associated with KEGG pathways are present throughout the entire genome, as shown in Fig. 1 for the pathways in the ‘Immune System’ group. This figure is typical for all the pathways that we could map genes to, and shows that the clustering of genes is different for each chromosome and pathway, with some pathways occupying long stretches of chromosomes. It should be noted that for a given pathway, SNPs associated with the pathway can be both in close proximity, at a great distance, or even on different chromosomes. This implies that selecting SNPs based on a single pathway will include information on SNPs from across the entire genome, some of which might be in high correlation with a causative locus.

For milk production, it has been shown that the gene *DGAT1* (diacylglycerol O-acyltransferase 1) contains a causative nucleotide [23], which violates our null hypothesis that all SNPs contribute equally to genetic variance, and thus, we expect that this will affect the outcome of the analyses. Due to linkage disequilibrium (LD), we expect that genes that are localized nearby to *DGAT1* will also have an effect. Thus, we arbitrarily defined that the genes located within 500 kbp of *DGAT1* constitute a group of genes, which we named ‘*DGAT1* genes’ (see Additional file 1: Table S1).

### Linear mixed model approach

By partitioning the markers into two groups, $\mathcal {S}$ and $\neg \mathcal {S}$, based on e.g. biological pathways or random gene groups, we used the following two-component linear mixed model to model the joint contribution of each set of markers. The model consists of two genetic effects, each modelled by a genomic relationship matrix, where each matrix is constructed from a set of markers. It can be written in linear form as:

(1) $$ \mathbf{y} = \mathbf{1}\mu + \mathbf{g}_{\mathcal{S}} + \mathbf{g}_{\neg\mathcal{S}} + \mathbf{e},   $$

where **y** is a 1×*n* vector of phenotypic observations, *n* the number of animals, *μ* the mean, $\mathbf {g}_{\mathcal {S}}$ is the *n* length vector of genetic effects for the markers in set $\mathcal {S}$, and similarly for $\neg \mathcal {S}$, and **e** the vector of residuals. The assumptions for the random effects are given by:

(2) $$  \begin{aligned} \left(\begin{array}{cc} \mathbf{g}_{\mathcal{S}} \\ \mathbf{g}_{\neg\mathcal{S}} \\ \mathbf{e} \end{array} \right) \sim N \left[\left(\begin{array}{ccc} \mathbf{0} \\ \mathbf{0} \\ \mathbf{0} \end{array} \right), \left(\begin{array}{ccc} \mathbf{G}_{\mathcal{S}}\sigma^{2}_{\mathcal{S}} & \mathbf{0} & \mathbf{0}\\ \mathbf{0} & \mathbf{G}_{\neg\mathcal{S}}\sigma^{2}_{\neg\mathcal{S}} & \mathbf{0} \\ \mathbf{0} & \mathbf{0} & \mathbf{D}{\sigma^{2}_{e}} \\ \end{array} \right) \right], \end{aligned}  $$

$\mathbf {g}_{\mathcal {S}}$ is the genomic relationship matrix, an *n*×*n* matrix, calculated as $\frac {{\mathbf {W}\mathbf {W}^{\prime }}}{m_{\mathcal {S}}}$, with **W** as the centered and scaled marker incidence matrix for the $m_{\mathcal {S}}$ markers in $\mathcal {S}$ [24].

$\mathbf {G}_{\neg \mathcal {S}}$ is similar, except for markers in $\neg \mathcal {S}$. **D** is the matrix of weights for the residuals, here a *n*×*n* diagonal matrix. For the health traits, this was equal to the identity matrix, and for the production traits, the diagonal was equal to the weights calculated as $\frac {r^{2}}{1-r^{2}}$, where *r* is the reliability of the DRP. $\sigma ^{2}_{\mathcal {S}}$, $\sigma ^{2}_{\neg \mathcal {S}}$, and ${\sigma ^{2}_{e}}$ are the variance components to be estimated for the genetic values and residuals, respectively.

A genomic relationship matrix constructed from a set of markers can describe pedigree-based relationships, i.e. the family structure [24]. In our case, $\mathbf {G}_{\neg \mathcal {S}}$ is always constructed based on at least 90 % of the markers, thus we expect that the population structure is always described [25, 26].

Under the infinitesimal model, it is assumed that all markers are equal when modelling their contribution to the genetic variance. The above model can therefore be reduced to a ‘simple model’ which serves as the competitive null hypothesis that variance is spread equally across all markers. Using the same notation as above, the simple model can be written as:

(3) $$ \mathbf{y} = \mathbf{1}\mu + \mathbf{g} + \mathbf{e},   $$

with assumptions as:

(4) $$ \begin{aligned} \left(\begin{array}{c} \mathbf{g} \\ \mathbf{e} \end{array} \right) \sim N \left[\left(\begin{array}{c} \mathbf{0} \\ \mathbf{0} \end{array} \right), \left(\begin{array}{cc} \mathbf{G} {\sigma^{2}_{g}} & \mathbf{0} \\ \mathbf{0} & \mathbf{D} {\sigma^{2}_{e}} \end{array} \right) \right], \end{aligned}  $$

which is identical to (1) when ${\sigma ^{2}_{g}} / m = \sigma ^{2}_{\mathcal {S}}/m_{\mathcal {S}} = \sigma ^{2}_{\neg \mathcal {S}} / m_{\neg \mathcal {S}}$,

(where *m* is the total number of markers). Thus the two models are nested. A proof of this follows by rewriting $\mathbf {G} = \frac {\mathbf { W}\mathbf {W}'}{m}$, and likewise for $\mathbf {g}_{\mathcal {S}}$ and $\mathbf {G}_{\neg \mathcal {S}}$ on their respective subset of markers.

(5) $$\begin{array}{*{20}l} \mathbf{G} {\sigma^{2}_{g}} &= \mathbf{G}_{\mathcal{S}} {\sigma^{2}_{g}} \frac{m_{\mathcal{S}}}{m} + \mathbf{G}_{\neg\mathcal{S}} {\sigma^{2}_{g}} \frac{m_{\neg\mathcal{S}}}{m} \notag \\ \frac{\mathbf{WW'}}{m} {\sigma^{2}_{g}} &= \frac{\mathbf{W}_{\mathcal{S}} \mathbf{W}'_{\mathcal{S}}}{m_{\mathcal{S}}} {\sigma^{2}_{g}} \frac{m_{\mathcal{S}}}{m} + \frac{\mathbf{W}_{\neg\mathcal{S}} \mathbf{W}_{\neg\mathcal{S}}'}{m_{\neg\mathcal{S}}} {\sigma^{2}_{g}} \frac{m_{\neg\mathcal{S}}}{m}  \\ \frac{\mathbf{WW'}}{m} {\sigma^{2}_{g}} &= \frac{\mathbf{W}_{\mathcal{S}} \mathbf{W}'_{\mathcal{S}} + \mathbf{W}_{\neg\mathcal{S}} \mathbf{W}_{\neg\mathcal{S}}'}{m} {\sigma^{2}_{g}}. \notag \end{array} $$

This notation makes it clear that the null-hypothesis does not assume that $\sigma ^{2}_{\mathcal {S}} = 0$, but rather that the variance components are equal when weighted by the respective proportions of SNPs that they represent.

We can relate the model in (1) to the model often used in GWAS, where the set $\mathcal {S}$ contains only a single marker:

$$ \mathbf{y} = \mathbf{1}\mu + \mathbf{g}_{\mathcal{S}} + \mathbf{g} + \mathbf{e}, $$

where $\mathbf {g}_{\mathcal {S}} \sim N \left (\mathbf {0}, \frac {\mathbf {W}_{\mathcal {S}} \mathbf {W}'_{\mathcal {S}}}{m_{\mathcal {S}}} \sigma ^{2}_{\mathcal {S}} \right)$ and $\mathbf {g} \sim N \left (\mathbf {0}, \frac {\mathbf {W}\mathbf {W}'}{m} {\sigma ^{2}_{g}} \right)$, i.e. the latter contains all markers. The corresponding null model for this model is the same as our null model in (3) and a standard LRT will test whether $\sigma ^{2}_{\mathcal {S}} > 0$. The GWAS model is similar to our model since it also attempts to capture the effect of a part of the genome, but differs in that the marker examined is included twice: i.e. both in **g**_*S*_, and in **g** via the construction of **G**. In our approach, the marker is only included once, and according to (5), LRT results should be easily interpreted. Recently, Yang et al. [27] showed that partitioning markers increased power to detect single marker associations and Speed and Balding [28] applied partitioning in MultiBLUP, which includes multiple random effects, each modelled by a subset of markers.

#### Estimation of parameters

The variance components were estimated using an averaged information restricted maximum likelihood (AI-REML) [29, 30] in the software DMU [31]. Special care is necessary to interpret the results of the AI-REML algorithm since it sometimes converges on a meaningless result; this happens when a variance component is close to zero, the likelihood ratio is negative, or as we found, the algorithm estimated 4 times more variance than present in the data. Our results were visually inspected for these shortcomings; less than 10 of more than 5500 random gene groups in each trait displayed this type of behaviour and were subsequently removed from the results, as well as one or two pathways for each of the production traits.

The proportion of explained genomic variance by a gene group $\mathcal {S}$, was calculated as:

(6) $$ \mathrm{H}^{2}_{\text{set}} = \frac{\sigma^{2}_{\mathcal{S}}}{\sigma^{2}_{\mathcal{S}} + \sigma^{2}_{\neg\mathcal{S}}}.   $$

*A priori*, all markers are expected to explain an equal amount of observed variance. Therefore, the ratio of expected variance for any marker group $\mathcal {S}$ with $m_{\mathcal {S}}$ markers from a total of *m* markers is expected to account for a proportion $m_{\mathcal {S}} / m$ of the observed genomic variance.

#### Likelihood ratio tests

The linear mixed model approach allows us to use a LRT to compare different pathway-based partitionings of the genomic variance. Likelihood ratios (LR) were calculated as twice the difference between the log-transformed likelihood of the simple model (3) and the full model (1). Standard theory describes that the LRT statistic used for this is distributed as $\chi ^{2}_{\kappa }$, where *κ* is the number of degrees of freedom and is equal to the difference in number of parameters between models [32]. When partitioning markers into two groups, given the chromosomal position of the markers, we expect the two groups to be correlated, although the model in (2) states that they are not. To what extent this partitioning influences the distribution of the LR is unclear. Thus, we determined the properties of LRT under these non-standard conditions using the random gene sampling procedure described below. This procedure will also take variation in the properties of groups into account, such as differences in allele frequencies, number of markers, and extent of LD, without attempting to model these properties explicitly.

### Random gene groups for empirical distributions

We applied a random sampling procedure to generate data under the competitive null hypothesis [33] that all markers are expected to account for the same amount of variance. This procedure differs greatly from a permutation test, which is typically used for a self-contained null hypothesis, in which a marker is expected to not account for any variance.

For each random gene group, a target number of markers was drawn uniformly within the range from 1 to 50 000. Genes were then sampled uniformly without replacement, until the total number of unique markers associated with these genes exceeded the target number. Variance components were estimated for each random gene group for each trait, resulting in more than 5500 data-points for each trait (see Table 1), each data-point consisting of the number of markers in the group (‘group size’), the ratio of variance explained by that group ($\mathrm {H}^{2}_{\text {set}}$ – see eq. 6), and the LR statistic.

An empirical threshold for $\mathrm {H}^{2}_{\text {set}}$ and LR was then calculated for each trait as a function of the number of markers by applying a smooth quantile regression from the R-package ‘quantreg’ [34–36] on the random gene groups at the 50th and 95th percentile, setting *λ*=500 and the constraint as ‘increasing’. The empirical thresholds are referred to as $H^{2}_{95}$ and *LR*_95_.

#### Correcting for multiple testing and false discovery rate

Based on empirical distributions of LR statistics, we applied a classical *χ*^2^ test of the LR statistics on each KEGG pathway with either 1 or 2 degrees of freedom (cf. results from Kolmogorov-Smirnov test). With p-values obtained from the *χ*^2^ distribution, the false discovery rate (FDR) was adjusted using the Benjamini and Hochberg method [37] using the ’p.adjust’-function in R v. 3.0.2 [38].

## Results

### Likelihood ratios

**Fig. 2:**
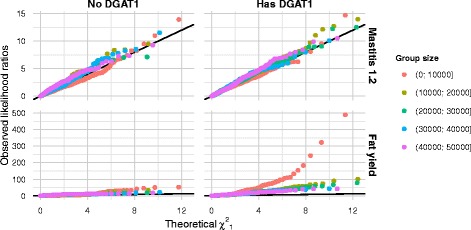
QQ-plot of the observed likelihood ratios (LR) of random gene groups vs. theoretical ${\chi ^{2}_{1}}$-distribution showing that they are skewed towards higher values than ${\chi ^{2}_{1}}$. LR displayed for the traits Mastitis 1.1 (top) and Fat yield (bottom), conditional on whether the gene groups contain one of the *DGAT1* genes (left/right) and group size (colour). Please note that the range of the y-axes differs between the top and bottom

Distributions of LR statistics are compared in Fig. 2 where the random gene groups are presented according to group size in intervals of 10000 markers. This revealed that the distributions for group sizes smaller than 10 000 markers differed in a trait-dependent way. For fat yield, the LR distribution was skewed towards larger values, whereas the LR distribution for health traits was slightly skewed towards smaller values, when the gene group included one of the *DGAT1* genes. We also found a weak, but significant increase in the 95th percentile of the LR statistics for different group sizes. Therefore, to better account for the influence of group size in the LRT, we used the quantile regression approach [34–36] to determine a 95 % cut-off adjusted for group size and the *DGAT1* genes in the gene groups.

We used the Kolmogorov-Smirnov test statistic to compare the empirical LR distributions to a *χ*^2^ distribution (see Additional file 2: Figure S1) and found that all traits, except two, had a smaller Kolmogorov-Smirnov test statistic for a *χ*^2^ distribution with one degree of freedom, than two degrees of freedom or a mixture of one and two degrees of freedom. I.e. the empirical LR distributions were more similar to a *χ*^2^ distribution with one degree of freedom. The two exceptions were ‘Fat yield’, which had a smaller test statistic for a *χ*^2^ distribution with two degrees of freedom, and ‘Milk yield’, which had the smallest test statistic for a mixture of the two *χ*^2^ distributions.

### Proportion of genomic variance explained

To illustrate how much of the genomic variance was explained by a randomly sampled group of genes, results for two trait are presented: a health trait (Mastitis 1.2 in Fig. 3) and a milk production trait (Fat yield in Fig. 4). Similar plots for all traits are in Additional file 2: Figures S2, S3, S4, S5, S6, S7, and S8.

**Fig. 3:**
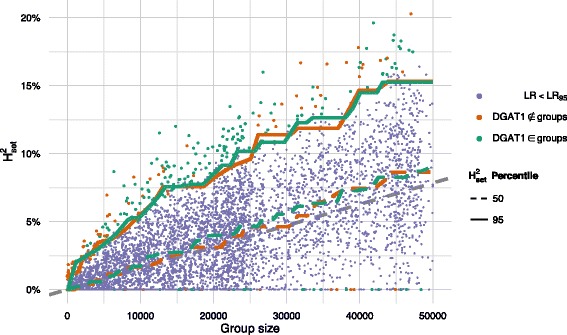
Proportion of explained genomic variance by random gene groups for the trait Mastitis 1.2 as a function of number of markers in the gene groups, showing that an increase in group size increases the expected amount of explained genomic variance. The dots corresponds to a random gene group, and the lines are the 50th and 95th percentile of these. The random gene groups are colour coded according to whether the likelihood ratio is larger than 95 % of the likelihood ratios of the same trait. The regression lines are coloured according to whether they describe gene groups containing *DGAT1* genes; the grey, dashed line corresponds to the naïve expectation of the infinitesimal model, where all markers contribute with the same effect

**Fig. 4:**
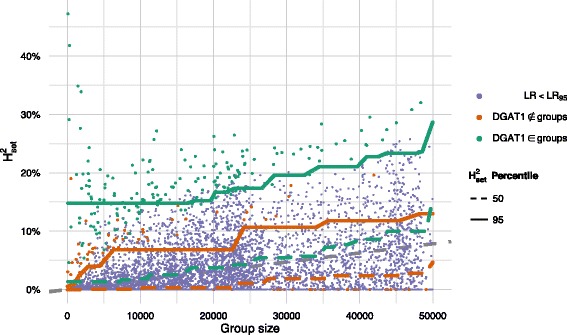
Proportion of explained genomic variance by random gene groups for the trait Fat yield as a function of number of markers in the gene groups, showing that groups with *DGAT1* genes consistently increase the expected amount of explained genomic variance. For groups that do not contain one of the *DGAT1* genes, the situation is the same as for Mastitis 1.2. The dots corresponds to a random gene group, and the lines are the 50th and 95th percentile of these. The random gene groups are colour coded according to whether the likelihood ratio is larger than 95 % of the likelihood ratios of the same trait. The regression lines are coloured according to whether they describe gene groups containing *DGAT1* genes; the grey, dashed line corresponds to the naïve expectation of the infinitesimal model, where all markers contribute with the same effect

In Figs. 3 and 4, the 50th and 95th percentiles for the proportion of genomic variance explained by groups of random genes clearly show an increase with increasing group size. The maximum amount of genomic variance explained by any gene group amounted to about 25 % and 50 % for milk yield and fat yield, respectively, whereas for protein yield and health traits, the maximum was 22 *%*. For the production traits, fat and milk yield, the influence of *DGAT1* on the amount of genomic variance explained was clear, since there were large differences in the $\mathrm {H}^{2}_{\text {set}}$-percentiles. For protein yield and the health traits, there was no difference between the regression lines for gene groups with *DGAT1* genes and gene groups without *DGAT1* genes, since this gene is not causative for these traits.

In Figs. 3 and 4, samples with LR statistics greater than the 95 % percentile (i.e. *LR*≥*LR*_95_) are highlighted, and in general, we found that gene groups that explained a large proportion of genomic variance also provided a better model fit. There was however a number of gene groups, that did not explain any of the variance, but still provided a good model fit.

### Criteria to determine the significance of a given gene group

Based on the empirical distribution of the test statistics from the random gene sampling procedure described above, we used two criteria to determine if a given pathway had a statistical significant better model fit or explained more variance than expected:

- *LR*_95_: Comparison of model fit. When *LR*≥*LR*_95_, group $\mathcal {S}$ had a better fit than 95 % of the random gene groups.
- ${H^{2}_{95}}$: Estimated proportion of explained genomic variance by markers in $\mathcal {S}$ exceeded that observed for 95 % of random gene groups of similar size. Our interpretation is that when $\mathrm {H}^{2}_{\text {set}} \geq H^{2}_{95}$, $\mathrm {H}^{2}_{\text {set}}$ is larger than expected by chance for a group with the same number of markers.

If both criteria were fulfilled, the partitioning of the subset $\mathcal {S}$ provided both a better model fit and explained more genomic variance than a random gene group, and thereby identified a gene group of particular biological interest. Both criteria are based on a competitive test, as in ‘Could we have found this result by randomly picking genes?’ and is in principle different from testing whether a variance component is different from zero (i.e. different from a self-contained test).

Using these criteria, it is not possible to correct for multiple testing, since neither *LR*_95_ nor $H^{2}_{95}$ provide p-values, but are merely cut-off values. However, the similarity between the theoretical *χ*^2^ and empirical distributions allows for the use of a classical *χ*^2^ test with accompanying adjustment of p-values, as demonstrated in the following.

### Biological pathways associated with health and production traits in dairy cattle

Some KEGG pathways could not be mapped to the bovine genome, or the AI-REML algorithm did not converge satisfactory, which resulted in LR statistics and estimates of genomic variance explained in between 148 and 150 gene groups, see rightmost column of Table 1. Table 2 summarizes how many of these KEGG pathways satisfied the two criteria (*LR*_95_ and $H^{2}_{95}$); generally, more pathways were found to pass *LR*_95_ than $H^{2}_{95}$. For each trait, 5 % (for ‘Somatic Cell Score’) to 12 % (for ‘Mastitis 1.1’) of the KEGG pathways met both criteria. The health trait ‘Somatic Cell Score’ consistently had the smallest proportion of explained genomic variance compared to the other traits, which was also evident for the associated pathways.

**Table 2:** Summary of significant KEGG pathways and combined pathways

		Number of pathways to pass			Combined pathways		Number of pathways to pass		
		$H^{2}_{95}$	*L* *R*_95_	Both	Number of SNPs	$H^{2}_{\textit {set}}$		*χ* ^2^	FDR
*Health traits*									
	Mastitis 1.1	20	20	18	8590	12.9		13	3
	Mastitis 1.2	10	18	9	7959	11.1		12	0
	Somatic Cell Score	11	8	8	2757	3.6		3	0
	Udder-health	17	22	16	11514	13.7		14	1
*Production traits*									
	Fat yield	14	16	14	7639	19.7		11	8
	Milk yield	15	19	14	10695	11.2		10	8
	Protein yield	14	23	13	12076	9.8		19	5

The ‘Combined pathways’ combine all markers associated with pathways found significant for each trait into a single pathway. All seven ‘Combined pathways’ were found significant by both *L* *R*_95_ and $H^{2}_{95}$. $H^{2}_{\textit {set}}$: Proportion of explained genomic variance. $H^{2}_{95}$ and *L* *R*_95_: Empirical cut-offs for $H^{2}_{\textit {set}}$ and LR. FDR: Benjamini and Hochberg p-value adjustment

Using the classical *χ*^2^ test, p-values could be calculated using the closest *χ*^2^ distribution from the Kolmogorov-Smirnov test, and adjusted for multiple testing. The number of pathways with a significant LR using a 10 % FDR is displayed in the rightmost column of Table 2. The number of pathways that were significant with an unadjusted *χ*^2^ test is similar to that obtained when using both empirically derived criteria.

**Fig. 5:**
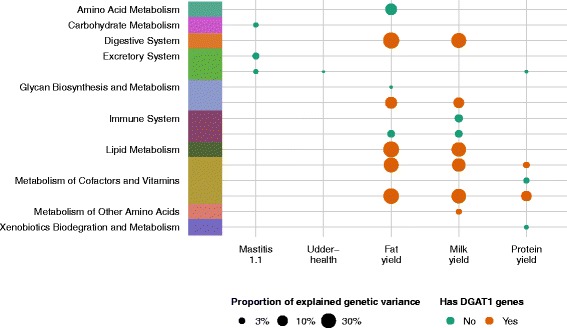
Overview of all pathways significant for both empirical cut-offs of LR and $\text {H}^{2}_{\text {set}}$ (*L* *R*_95_ and $H^{2}_{95}$), which shows that some pathways are consistently significant for multiple traits. Pathways are colour coded by group, points are sized by proportion of explained genomic variance ($\text {H}^{2}_{\text {set}}$)

Figure 5 summarises the pathways that have a significant LR after adjusting for multiple testing using FDR. The values for $\mathrm {H}^{2}_{\text {set}}$, adjusted p-value, and LR statistics for these pathways are in Table 3. After correcting for multiple testing, there were no pathways associated with ‘Mastitis 1.2’ or ‘Somatic Cell Count’. A summary of all tested pathways and traits is in Table S2 (see Additional file 3: Table S2).

**Table 3:** Values of significant KEGG pathways, after adjusting for multiple testing

	Pathway	Trait	Number of SNPs	$\text {H}^{2}_{\text {set}}$	p-value	LR
*Amino Acid Metabolism*						
	Arginine and proline metabolism	Fat yield	390	13.8 %	6·10^−9^	44.3
*Carbohydrate Metabolism*						
	Pentose phosphate pathway	Mastitis 1.1	182	2.3 %	4·10^−2^	11.6
*Digestive System*						
	Fat digestion and absorption	Fat yield	436	43.4 %	0	317
	Fat digestion and absorption	Milk yield	436	31.5 %	0	184
*Excretory System*						
	Excretory System	Mastitis 1.1	1639	3.6 %	4·10^−2^	11.2
	Proximal tubule bicarbonate reclamation	Udder- health	187	1.2 %	8·10^−2^	11.9
	Proximal tubule bicarbonate reclamation	Mastitis 1.1	187	2.2 %	1·10^−4^	24.1
	Proximal tubule bicarbonate reclamation	Protein yield	187	1.5 %	6·10^−2^	10.8
*Glycan Biosynthesis and Metabolism*						
	Glycosphingolipid biosynthesis - globo series	Fat yield	91	1.4 %	3·10^−2^	13.2
	Glycosylphosphatidylinositol(GPI)-anchor biosynthesis	Fat yield	157	14.4 %	0	91.6
	Glycosylphosphatidylinositol(GPI)-anchor biosynthesis	Milk yield	157	10.3 %	2·10^−12^	60.6
*Immune System*						
	Chemokine signaling pathway	Milk yield	2809	5.3 %	3·10^−3^	16.8
	Leukocyte transendothelial migration	Milk yield	2001	4.6 %	5·10^−3^	15.6
	Leukocyte transendothelial migration	Fat yield	2001	4.5 %	6·10^−3^	16.4
*Lipid Metabolism*						
	Glycerolipid metabolism	Milk yield	969	27.9 %	7·10^−12^	57.4
	Glycerolipid metabolism	Fat yield	969	41.6 %	0	157
*Metabolism of Cofactors and Vitamins*						
	Metabolism of Cofactors and Vitamins	Milk yield	1796	21.9 %	4·10^−9^	44.5
	Metabolism of Cofactors and Vitamins	Fat yield	1796	32.0 %	3·10^−12^	59.7
	Metabolism of Cofactors and Vitamins	Protein yield	1796	3.4 %	8·10^−2^	9.5
	Porphyrin and chlorophyll metabolism	Protein yield	194	3.0 %	6·10^−2^	10.4
	Retinol metabolism	Fat yield	231	38.1 %	0	382
	Retinol metabolism	Milk yield	231	28.9 %	0	259
	Retinol metabolism	Protein yield	231	9.9 %	2·10^−9^	45.5
*Metabolism of Other Amino Acids*						
	Glutathione metabolism	Milk yield	231	2.9 %	3·10^−2^	12
*Xenobiotics Biodegration and Metabolism*						
	Xenobiotics Biodegration and Metabolism	Protein yield	860	1.9 %	8·10^−2^	9.04

Displayed p-values are adjusted

Two of the pathway groups were significant; ‘Excretory System’ and ‘Metabolism of Cofactors and Vitamins’. The pathway ‘Retinol metabolism’, which includes *DGAT1*, is significant for all three production traits; it passes the two criteria *LR*_95_ and $H^{2}_{95}$ for all traits (except ‘Mastitis 1.1’, which was only found significant by $H^{2}_{95}$).

There is almost no overlap between pathways that were significant for production traits and health traits. Only the pathway ‘Proximal tubule bicarbonate reclamation’ is significant for both ‘Udder-health’ and ‘Protein yield’, and explains about the same amount of genomic variance in the two traits.

## Discussion

A LMM and a statistical testing strategy were presented to examine the joint contribution of a marker set to a complex trait, where the markers are located in gene groups associated to a biological pathway.

Our LMM was based on partitioning the genomic variance into two components. One component due to the markers in a gene group and another component due to the remaining set of markers. The model was similar to those proposed by Jensen et al. [11] and Listgarten et al. [26] who also used multiple genomic variance components. In their studies, they only investigated consecutive regions of markers sets, i.e. individual genes or chromosomes, whereas we examined scattered sets of markers; i.e. the biological pathways.

We used two approaches to evaluate a gene group: LRT and thresholds based on empirical distributions.

We used the LRT to evaluate the model fit of a gene group. A high LR showed that the model with two different genomic variance components performed better to explain the observed genomic variance than a model with only a single genomic variance component. It is important to note that this LRT was different from a standard LRT, which is used to test whether the variance component for a random effect is different from zero. Our null hypothesis corresponds to the infinitesimal model where all markers are - on average - contributing with the same, albeit small effect. This corresponds to our null model with a single genomic variance component. Our alternative hypothesis is that the causal markers are not randomly distributed over the genome, as in the null hypothesis, but are clustered in gene groups that can be associated to certain biological pathways. This is reflected in our alternative model with two genomic variance components. If the null hypothesis is true, we expect that the null and alternative models lead to a similar fit. If the alternative hypothesis is true, we expect that the alternative model to provide a better model fit. Furthermore, under the null hypothesis, we expect that the proportion of explained genomic variance by a gene group is proportional to the number markers (or genome length covered) by the gene group. Thus our LRT reflects the statistical significance of the partitioning of the genomic variance. However, a high LR alone does not indicate that a gene group is biologically important since we observed several gene groups with a high LR that explained close to none of the genomic variance. Thus, it was necessary to use both LR and proportions of explained genomic variance to identify biological important gene groups.

To estimate the expected proportion of explained genomic variance under the null hypothesis, we used a resampling approach. Here, loci were used as sampling units cf. the null hypothesis for which all markers were expected to contribute. Thus our resampling approach generated data under the null hypothesis. However, not all loci were sampled, since we restricted the analysis to sampling random gene groups to be able to compare with gene groups associated to biological pathways. By using the empirical distributions from the resampling approach, we derived cut-off thresholds for both LR and explained genomic variance. This was used to assess whether the observed estimates of LR or explained genomic variance for a biological pathway showed extreme values compared to those for a random gene group with a similar number of markers in this particular sample of Danish Holstein animals. The p-values obtained by the LRT are conceptually different from the thresholds obtained from the resampling described above. The p-values relate to true biological replication of the experiment. A significant p-value excludes random variation at the animal level as an explanation for the associations found, and therefore increases the confidence that the same associations will be found for a new sample of animals from the same breed. In spite of this different interpretation of the p-values, we observed a large overlap between the biological pathways that were determined to be ‘significant’ using either p-values obtained from the LRT or the thresholds obtained from the resampling procedure.

**Empirical distribution of likelihood ratios** The empirical LR distribution for protein yield and the health traits resembled a *χ*^2^ distribution with one degree of freedom, whereas it was closer to a *χ*^2^ distribution with two degrees of freedom for fat and milk yield. A consistent pattern across all traits was that the observed likelihood ratios were skewed towards higher values at the right hand tail of the empirical LR distribution, as compared to the theoretical *χ*^2^ distribution with one degree of freedom. This was not entirely unexpected since some of the random gene groups were likely to harbour markers that are linked to causal genetic variants. Examining the LR distributions, we could roughly divide the traits into two groups: fat and milk yields, which are strongly affected by *DGAT1*, and the health traits and protein yield, which are less affected by *DGAT1*. However, for the health traits and protein yield, there was also a clear effect of *DGAT1*, since the 95th percentile of the LR was significantly higher when *DGAT1* was included in the gene group. This could indicate that the region that includes *DGAT1* also harbours genetic variants that are associated with the health traits.

**Empirical distribution of proportion of explained genomic variance** Based on the infinitesimal model, we would expect that each marker contributes equally to the observed genomic variance. A gene group with *n* markers would be expected to account for *n*/*n*_*total*_ of the genomic variance. The median of explained genomic variance by the random gene groups followed this expected proportion. This can be considered as an argument in favour of the infinitesimal model. There were, however, numerous samples for which a small number of markers accounted for more of the genomic variance than expected. Overall, these deviations from the infinitesimal model are taken into account in our testing procedure, by using random gene samples.

### Biological pathways associated with udder health and production traits

We found that KEGG pathways of the categories ‘Carbohydrate Metabolism’ and ‘Excretory System’ were associated with the health traits, but not with the production traits (with the exception of protein yield). Production traits were strongly associated with – among others – the categories ‘Amino Acid Metabolism’, ‘Digestive System’, ‘Lipid Metabolism’, and ‘Metabolism of Cofactors and Vitamins’. Surprisingly, the category ‘Immune System’ was found to be associated with the production traits but with none of the health traits; together with ‘Arginine and proline metabolism’, the two ‘Immune System’ pathways are the only non-*DGAT1* pathways associated with the production traits.

The transcriptome of the mammary gland changes during the lactation cycle, and these modifications reflect its developmental and physiological activities at different stages. The results of a recent study by Bionaz et al. [39] on the cow mammary transcriptome during lactation provide a rationale for understanding several of the associations between KEGG pathways and the traits observed here. All categories that were found associated in our study, except ‘Xenobiotics Biodegration and Metabolism’, were also found in [39]. One of these shared categories was the ‘Lipid Metabolism’ pathway ‘Glycerolipid metabolism’. This was not entirely unexpected considering that *DGAT1* catalyses the final step of triacylglycerol synthesis, and that there is a strong association between fat content of milk and *DGAT1* polymorphisms [23]. Furthermore, differential gene expression during lactation also impacted ‘Retinol metabolism’ in the category ‘Metabolism of Cofactors and Vitamins’ [39], which probably reflects the contribution of retinoic acid signalling to mammary gland morphogenesis and function [40, 41]. Furthermore, it has been shown that retinol metabolism involves retinol esterification, which is catalyzed by *DGAT1*, and thus explains the striking influence of the *DGAT1* gene [42, 43] on fat yield.

Comparison of the gene expression patterns in the mammary gland tissue of cows with and without mastitis has been used to identify genes and pathways that have a role in host defense against infection [44]. Intersection of the pathways in the present data and in the infection-based data revealed a marked overlap, including the pathways ‘Chemokine signalling’, ‘Leukocyte transendothelial migration’, ‘Retinol metabolism’, and ‘Glycerolipid metabolism’. Interestingly, we observed that these KEGG pathways were associated with production traits rather than with health traits, which might reflect the negative correlation between mastitis resistance and milk production. However a multiple trait variance decomposition analysis of mastistis and milk production traits is required to disentangle the negative genetic correlation. The overlap between KEGG pathways presented here and those derived from transcriptomes of infected or lactating mammary gland lend support to the present genome partitioning approach as a means of understanding the genetics of complex traits.

The discussion of these pathways is not meant to be exhaustive but rather to give examples of the power of our approach to reveal biologically meaningful pathways and phenotypic traits.

Our LMM approach has several advantages. First, it builds on a solid statistical modelling framework that allows us to adjust for other known effects, such as gender, age, and various lifestyle characteristics, and to use it in populations with complex pedigree structures. Second, it can be easily extended to handle multiple correlated traits and thus be used to identify biological pathways that underlie the genetic correlations. From a selective breeding perspective, it would be very useful if this information could be used to design selection strategies that limit the influence of unfavourable correlations between complex traits and diseases. Third, LMM are commonly applied to predict the genetic value (or risk) in genomic selection programs. Although our approach can be used to predict genetic values based on each marker set, further work is required to assess if this would lead to more accurate predictions of genetic values.

Our findings not only improve our understanding of the biological basis of traits but also provide a source of molecular information on candidate genes and pathways that, when applied in breeding programs, could lead to higher precision in the prediction of genetic values and allow faster progress towards breeding goals.

## Conclusions

In this study, we used a LMM approach to examine the joint contribution of genetic markers associated with a biological pathway to a complex trait. The random gene sampling procedure allowed us to assess the statistical significance of the estimated variance explained by the biological pathways. This was done with a dataset that has an inherently complex correlation structure due to the population structure, and LD within the bovine genome and the gene group itself. We identified several biological pathways that were significant for both health traits and milk production traits; thus, we show that the markers associated to these pathways explain more of the genomic variance and provide a better model fit than 95 % of randomly sampled gene groups.

We believe that the linear mixed model approach provides a general framework to exploit and integrate multiple layers of data from high throughput genome technologies, potentially leading to improved understanding of the genetic architecture of complex traits and diseases.

## Additional files

Additional file 1
**Table S1 of ‘** ***DGAT1*** **genes’.** Genes included in the set of ‘*DGAT* genes’, alongside their chromosomal position, gene symbol, and Entrez gene ID.

Additional file 2
**Figures S1 through S8.** Figure S1: Kolmogorov-Smirnov test statistics; Figure S2 through S8, plots of group size vs. $\text {H}^{2}_{\text {set}}$ for all seven traits.

Additional file 3
**Table S2 with overview of all tested pathways.** All test statistics (LRT, $\text {H}^{2}_{\text {set}}$, p-values, size, variance components, etc.) of tested pathways. Description of columns are in file.

## References

[1] de los Campos G, Gianola D, Allison DB (2010). Predicting genetic predisposition in humans: the promise of whole-genome markers. Nat Rev Genet.

[2] Manolio TA, Collins FS, Cox NJ, Goldstein DB, Hindorff LA, Hunter DJ (2009). Finding the missing heritability of complex diseases. Nature.

[3] Makowsky R, Pajewski NM, Klimentidis YC, Vazquez AI, Duarte CW, Allison DB (2011). Beyond missing heritability: prediction of complex traits. PLoS Genet.

[4] Yang J, Benyamin B, McEvoy BP, Gordon S, Henders AK, Nyholt DR (2010). Common SNPs explain a large proportion of the heritability for human height. Nat Genet..

[5] Lango AH, Estrada K, Lettre G, Berndt SI, Weedon MN, Rivadeneira F, Willer CJ (2010). Hundreds of variants clustered in genomic loci and biological pathways affect human height. Nature.

[6] Peñagaricano F, Weigel KA, Rosa GJM, Khatib H (2012). Inferring quantitative trait pathways associated with bull fertility from a genome-wide association study. Front Genet.

[7] Wang K, Li M, Hakonarson H (2010). Analysing biological pathways in genome-wide association studies. Nat Rev Genet.

[8] Wu MC, Lee S, Cai T, Li Y, Boehnke M, Lin X (2011). Rare-variant association testing for sequencing data with the sequence kernel association test. Am J Hum Genet..

[9] Silver M, Montana G (2012). Fast identification of biological pathways associated with a quantitative trait using group lasso with overlaps. Stat Appl Genet Molec Biol.

[10] Fridley BL, Biernacka JM (2011). Gene set analysis of SNP data: benefits, challenges, and future directions. Eur J Hum Genet..

[11] Jensen J, Su G, Madsen P (2012). Partitioning additive genetic variance into genomic and remaining polygenic components for complex traits in dairy cattle. BMC Genet..

[12] Kanehisa M, Goto S (2000). KEGG: Kyoto encyclopedia of genes and genomes. Nucleic Acids Res..

[13] Kanehisa M, Goto S, Sato Y, Furumichi M, Tanabe M (2012). KEGG for integration and interpretation of large-scale molecular data sets. Nucleic Acids Res..

[14] Browning BL, Browning SR (2009). A unified approach to genotype imputation and haplotype-phase inference for large data sets of trios and unrelated individuals. Am J Hum Genet..

[15] Su G, Brøndum RF, Ma P, Guldbrandtsen B, Aamand GP, Lund MS (2012). Comparison of genomic predictions using medium-density (∼ 54,000) and high-density (∼777,000) single nucleotide polymorphism marker panels in Nordic Holstein and Red Dairy Cattle populations. J Dairy Sci..

[16] NAV routine genetic evaluation of dairy cattle, 2nd edn.: Nordic Cattle Genetic Evaluation; 2013. http://www.nordicebv.info.

[17] Stranden I, Mantysaari EA (2010). A recipe for multiple trait deregression. Interbull Bull.

[18] Lidauer M, Strandén I (1999). Fast and flexible program for genetic evaluation in dairy cattle. International Workshop on High Performance Computing and New Statistical Methods in Dairy Cattle Breeding.

[19] Vuori K, Strandén I, Lidauer M, Mantysaari EA. MiX99 - Effective solver for large and complex linear mixed models. In: Proceeding of the 8^th^ World Congress on Genetics Applied to Livestock Production: 13–18 August 2006. Belo Horizonte, Brazil.

[20] Zimin AV, Delcher AL, Florea L, Kelley DR, Schatz MC, Puiu D (2009). A whole-genome assembly of the domestic cow, Bos taurus. Genome Biol..

[21] Gentleman R, Carey V, Bates D, Bolstad B, Dettling M, Dudoit S (2004). Bioconductor: open software development for computational biology and bioinformatics. Genome Biol..

[22] Carlson M, Falcon S, Pages H, Li N. org.Bt.eg.db: Genome wide annotation for Bovine. R package version 2.5.0. 2011. http://www.bioconductor.org/packages/2.8/data/annotation/html/org.Bt.eg.db.html Accessed September 1st 2011.

[23] Grisart B, Farnir F, Karim L, Cambisano N, Kim J-J, Kvasz A (2004). Genetic and functional confirmation of the causality of the DGAT1 K232A quantitative trait nucleotide in affecting milk yield and composition. Proc Natl Acad Sci U S A.

[24] VanRaden PM (2007). Genomic measures of relationship and inbreeding. Interbull Bull..

[25] Lippert C, Listgarten J, Liu Y, Kadie CM, Davidson RI, Heckerman D (2011). FaST linear mixed models for genome-wide association studies. Nat Methods.

[26] Listgarten J, Lippert C, Kang EY, Xiang J, Kadie CM, Heckerman D (2013). A powerful and efficient set test for genetic markers that handles confounders. Bioinformatics..

[27] Yang J, Zaitlen NA, Goddard ME, Visscher PM, Price AL (2014). Advantages and pitfalls in the application of mixed-model association methods. Nat Genet..

[28] Speed D, Balding DJ (2014). MultiBLUP: improved SNP-based prediction for complex traits. Genome Res..

[29] Madsen P, Jensen J, Thompson R. Estimation of (co)variance components by REML in multivariate mixed linear models using average of observed and expected information. In: Proceeding of the 5^th^ World Congress on Genetics Applied to Livestock Production: 7–12 August 1994. Guelph, ON, Canada. p. 455–62.

[30] Johnson DL, Thompson R (1995). Restricted maximum likelihood estimation of variance components for univariate animal models using sparse matrix techniques and average information. J Dairy Sci..

[31] Madsen P, Jensen J. A User’s Guide to DMU. A package for analysing multivariate mixed models. Version 6, release 5.1. Tjele, Denmark; 2012. http://dmu.agrsci.dk/DMU/Doc/Previous/dmuv6_guide.5.1.pdf Accessed December 1st 2012.

[32] Visscher PM (2006). A note on the asymptotic distribution of likelihood ratio tests to test variance components. Twin Res Hum Genet..

[33] Goeman JJ, Bühlmann P (2007). Analyzing gene expression data in terms of gene sets: methodological issues. Bioinformatics..

[34] Koenker R. quantreg: Quantile regression. 2012. http://cran.r-project.org/web/packages/quantreg/index.html Accessed October 17th 2012.

[35] Koenker R, Ng P, Portnoy S (1994). Quantile smoothing splines. Biometrika..

[36] Koenker R, Basset G (1978). Regression quantiles. Econometrica..

[37] Benjamini Y, Hochberg Y (1995). Controlling the false discovery rate: A practical and powerful approach to multiple testing. J Roy Statist Soc Ser B..

[38] R Core Team (2012). R: A language and environment for statistical computing.

[39] Bionaz M, Periasamy K, Rodriguez-Zas SL, Everts RE, Lewin HA, Hurley WL (2012). Old and new stories: Revelations from functional analysis of the bovine mammary transcriptome during the lactation cycle. PLoS ONE..

[40] Cho K-W, Kwon H-J, Shin J-O, Lee J-M, Cho S-W, Tickle C (2012). Retinoic acid signaling and the initiation of mammary gland development. Dev Biol..

[41] Wang YA, Shen K, Wang Y, Brooks SC (2005). Retinoic acid signaling is required for proper morphogenesis of mammary gland. Dev Dyn..

[42] O’Byrne SM, Blaner WS (2013). Retinol and retinyl esters: biochemistry and physiology. J Lipid Res..

[43] Shih MYS, Kane MA, Zhou P, Yen CLE, Streeper RS, Napoli JL (2009). Retinol esterification by DGAT1 is essential for retinoid homeostasis in murine skin. J Biol Chem..

[44] Buitenhuis B, Røntved CM, Edwards SM, Ingvartsen KL, Sørensen P (2011). In depth analysis of genes and pathways of the mammary gland involved in the pathogenesis of bovine Escherichia coli-mastitis. BMC Genomics..

