# Supplementary material for: Partitioning of genomic variance reveals biological pathways associated with udder health and milk production traits in dairy cattle
Source: Genet Sel Evol. 2015 Jul 14;47(1):60. doi: 10.1186/s12711-015-0132-6 (PMC4499908; doi:10.1186/s12711-015-0132-6)
Supplement: Additional file 2 — Figures S1 through S8. Figure S1: Kolmogorov-Smirnov test statistics; Figure S2 through S8, plots of group size vs. $\text {H}^{2}_{\text {set}}$Hset2 for all seven traits. [file 12711_2015_132_MOESM2_ESM.pdf]

Supplementary tables and figures

For 'Partitioning for genomic variance reveals biological pathways associated with udder health and milk production traits in dairy cattle'

Stefan McKinnon Edwards `stefan.hoj-edwards@mbg.au.dk`
Center of Quantitative Genetics and Genomics,
Dept. Molecular Biology and Genetics,
Aarhus University, Denmark

June 11, 2015

List of Figures

S1 Distance between observed likelihood ratios and theoretical χ^2 distributions. 1
S2 Mastitis 1.1 3
S3 Mastitis 1.2 4
S4 Somatic Cell Score 4
S5 Udder-health 5
S6 Fat yield 5
S7 Milk yield 6
S8 Protein yield 6

Kolmogorov-Smirnov Tests

Here, we would like to estimate the difference between our empirically estimated distributions and the χ^2 distributions more statistically, that is with the Kolmogorov-Smirnov test.

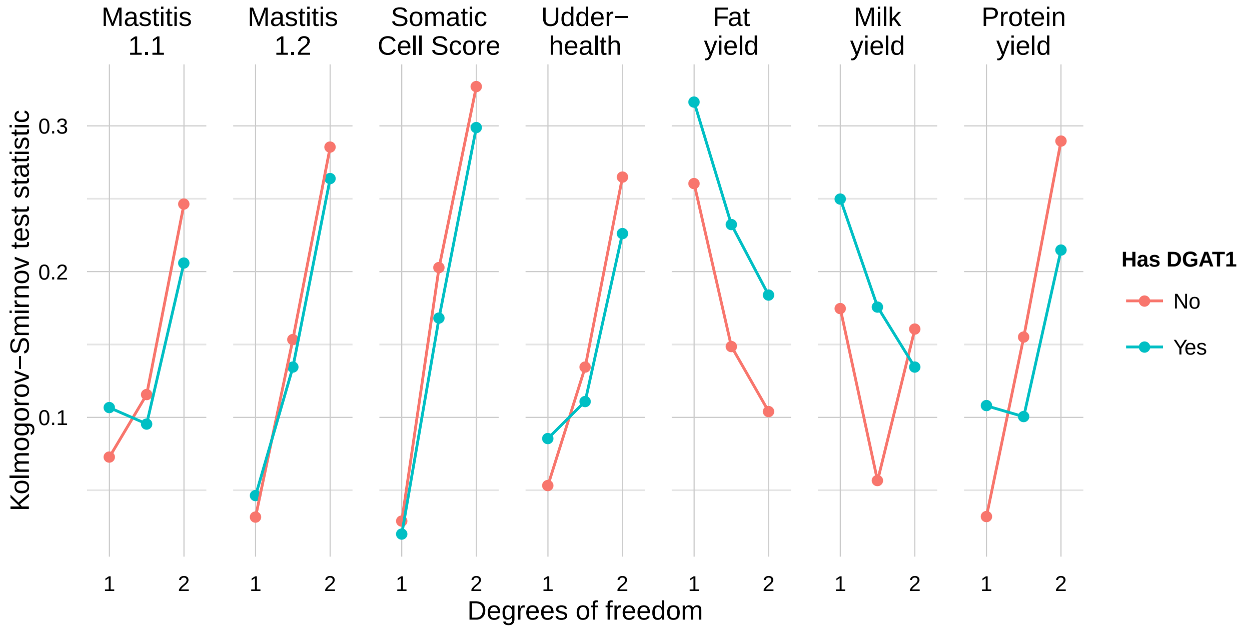

Figure S1: *Distance between observed likelihood ratios and theoretical* χ^2 *distributions.*

1 Group size vs. H^2_{set}

The figures in this section displays the proportion of explained genetic variance explained by random gene groups, as a function of the number of markers in the groups ('group size'), plotted together with the 50th and 95th percentile. The groups are colour coded by whether the likelihood ratio is larger than 95% of the likelihood ratios of the same trait. The regression lines are coloured by whether they describe groups containing DGAT1 genes; the grey, dashed line corresponds to the naïve expectation of the infinitesimal model, where all genetic markers contribute with the same effect.

1.1 Health traits

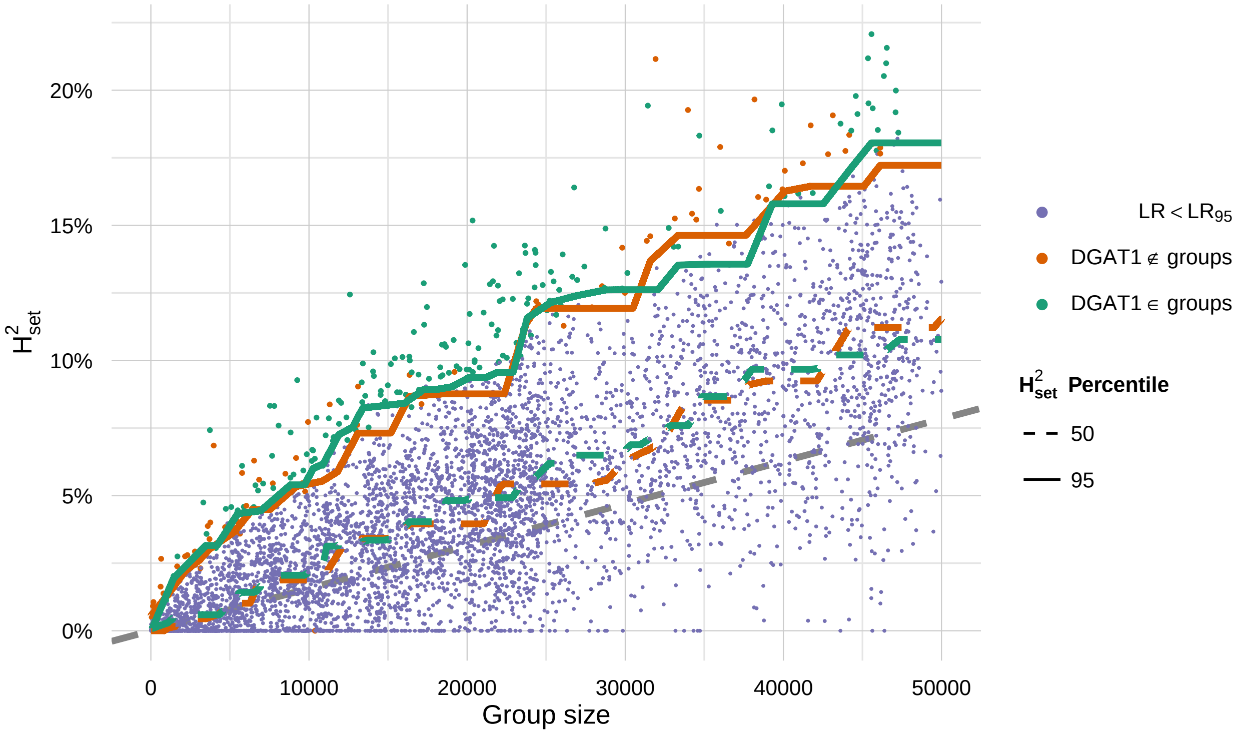

Figure S2: *Mastitis 1.1*

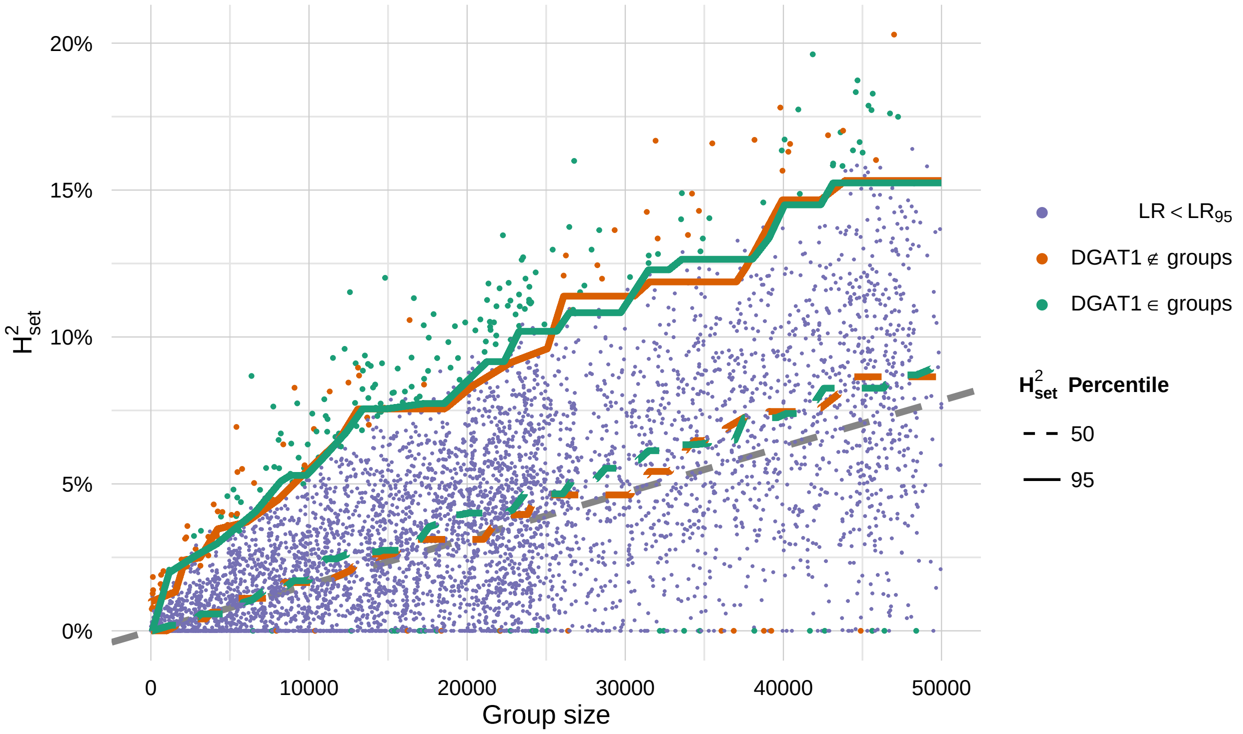

Figure S3: *Mastitis 1.2*

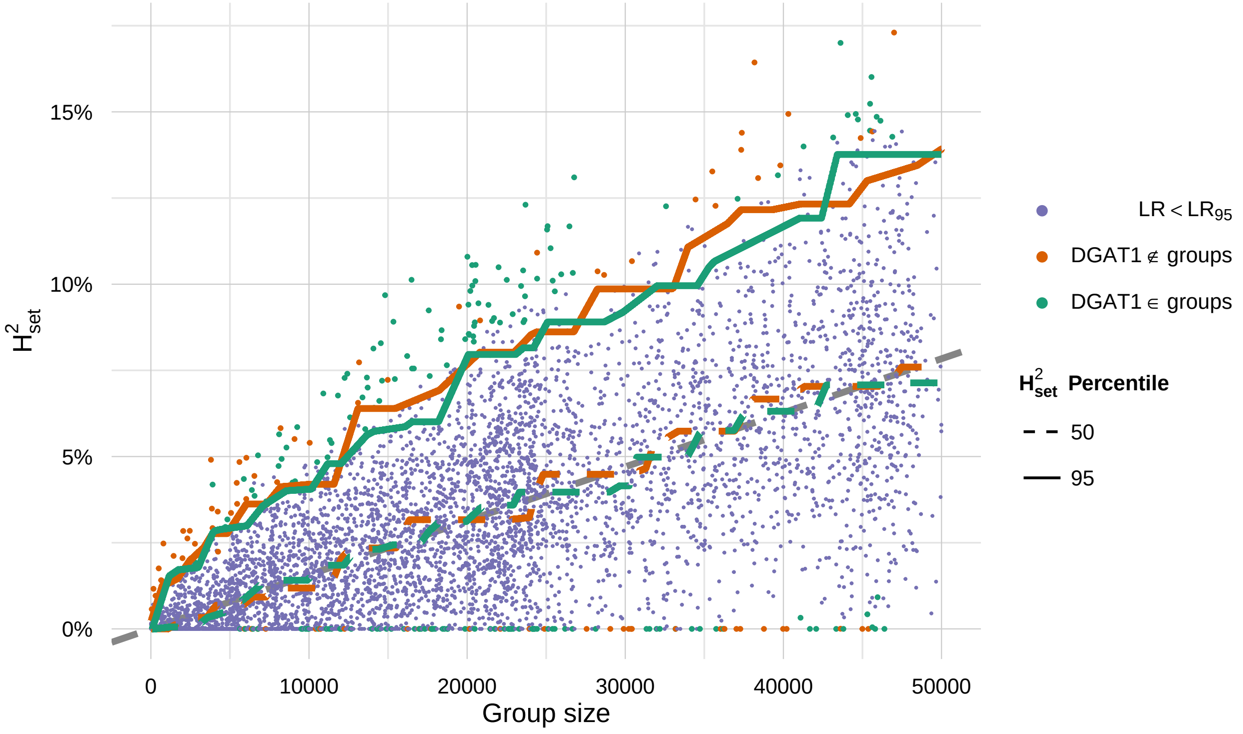

Figure S4: *Somatic Cell Score*

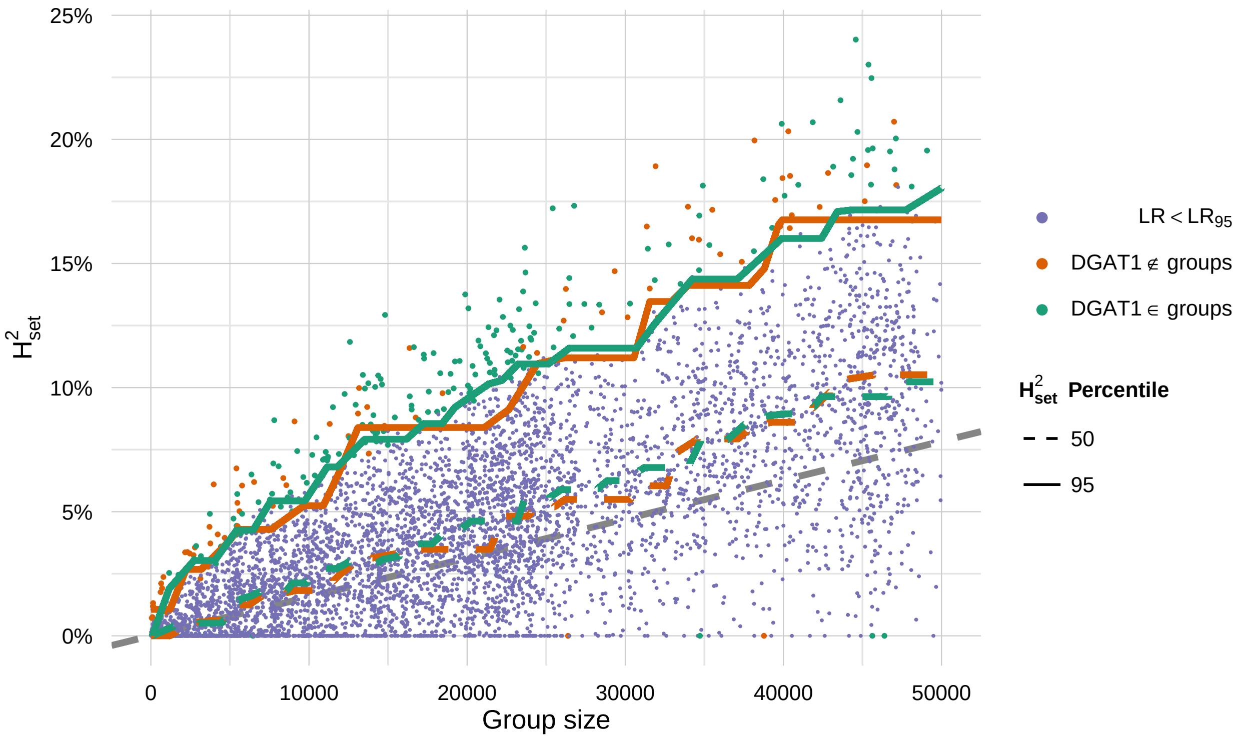

Figure S5: *Udder-health*

1.2 Production traits

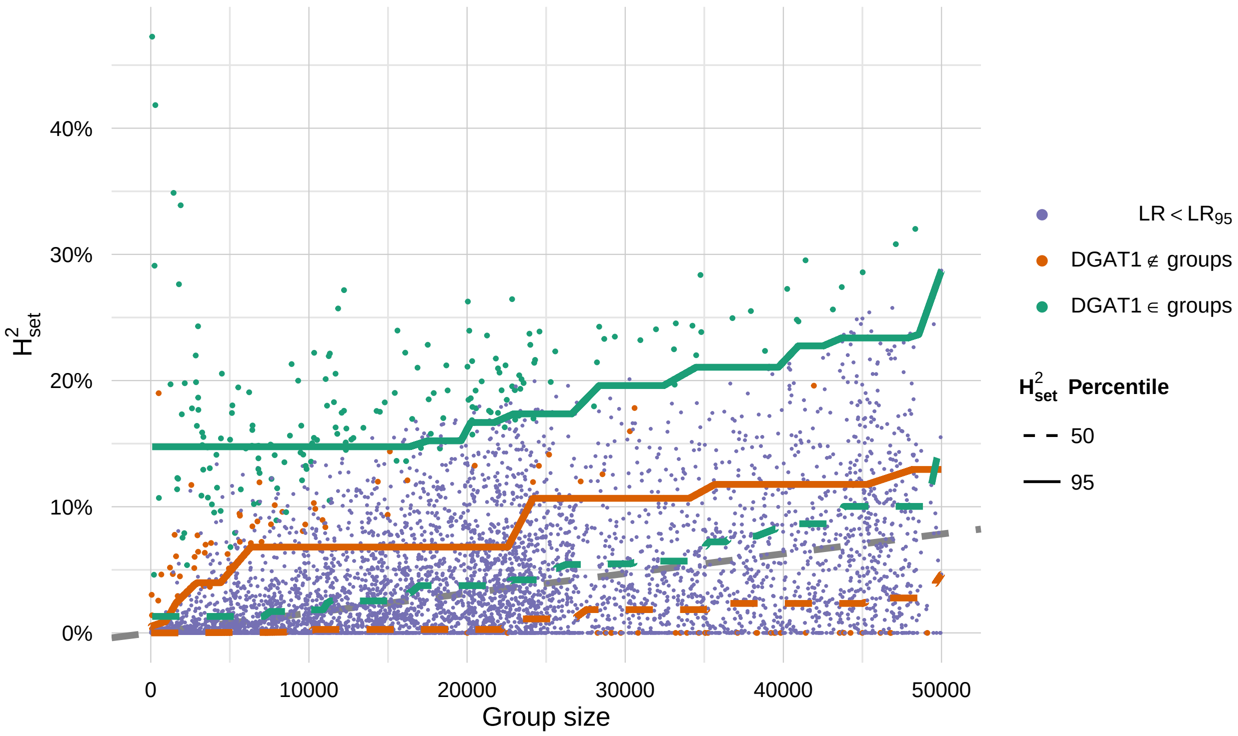

Figure S6: *Fat yield*

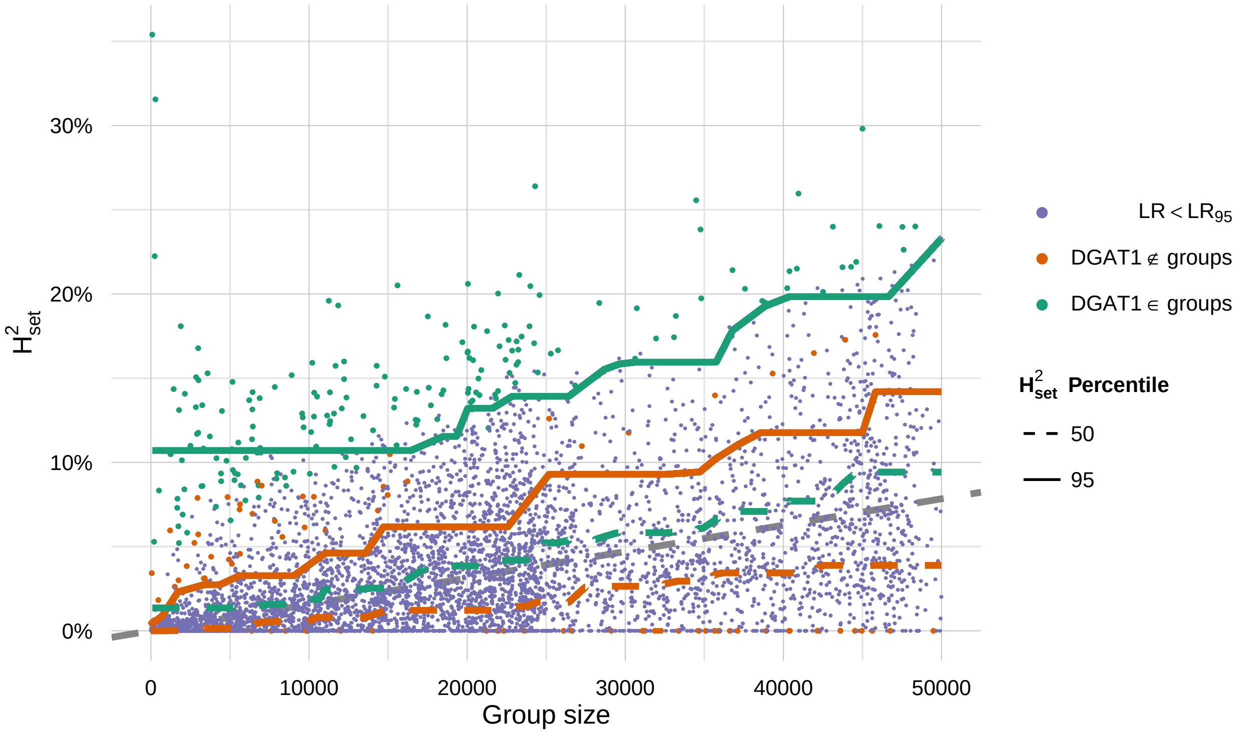

Figure S7: *Milk yield*

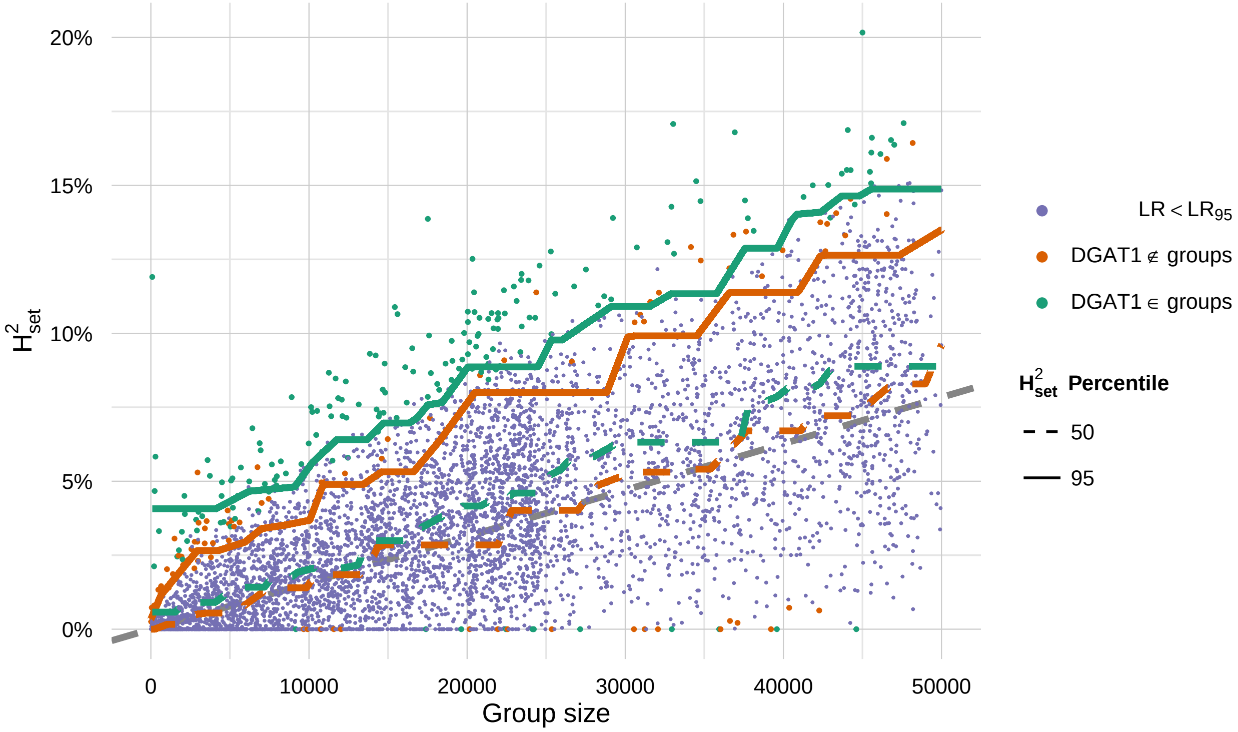

Figure S8: *Protein yield*
